# Differentiation of Ecological Niches in Trophic Specialists From a Disturbed Lacustrine Ecosystem: Insights from the Sympatric Lake Tana Labeobarbs (Cyprinidae)

**DOI:** 10.1002/ece3.73098

**Published:** 2026-02-13

**Authors:** Evgeny V. Esin, Anastasia D. Kudryavtseva, Boris A. Levin, Belay Abdissa, Benyam Hailu, Fedor N. Shkil, Axel Meyer

**Affiliations:** ^1^ A.N. Severtsov Institute of Ecology and Evolution RAS Moscow Russia; ^2^ Bahir Dar Fisheries and Other Aquatic Life Research Center Bahir Dar Ethiopia; ^3^ Chair in Zoology and Evolutionary Biology, Department of Biology University of Konstanz Konstanz Germany

**Keywords:** adaptive radiation, cyprinids, ecosystem impact, endangered species, lacustrine fish, trophic specialization

## Abstract

Adaptive radiations of freshwater fishes are based on the partitioning of trophic resources as sympatric forms diverge and end up occupying unique and specialized trophic niches. It remained unknown how these specialists responded to recent human‐induced ecosystem disturbances. Here, we tested how the species with various degrees of tropho‐ecological specializations (generalists vs. specialists) of Lake Tana *Labeobarbus* spp. flock reacted to the ongoing detrimental human‐induced ecosystem transformation and the resulting drastic population declines. In late 2022, we collected adults of the still remaining species to examine current niche partitioning within the assemblage based on the analyses of muscle stable isotope ratios and fatty acid compositions as time‐ and space‐integrated tropho‐ecological markers. The data revealed one niche for the generalized omnivorous labeobarb, four distinct niches for the nonpiscivorous, as well as three discernible niches for the piscivorous labeobarbs. Of the 11 species caught in the lake, only two pairs showed demonstrably large niche overlap. Of all the species in the Lake Tana species flock, the top (piscivorous) predators were the most strongly affected by the disturbance in ecological parameters, which resulted in altered and narrowed niches. This suggests that most of the endemic labeobarb species still retain their original, stable, and distinct diets and habitat preferences that appeared to have maintained the overall ecological relationships in Lake Tana. We also detected that some species apparently changed their diet, seemingly adapting to human‐induced habitat disturbances and large population declines during the last decades.

## Graphical abstract

Lake Tana harbors the world's most complex adaptive radiation of cyprinids in the world. Although trophic specialization has been proven to drive this diversification, the diet of endemics has been studied using only traditional stomach content analysis. Here we present a study of the trophic differentiation of Lake Tana labeobarbs based on the analysis of muscle stable isotope ratios and fatty acid compositions as the time‐ and space‐integrated tropho‐ecological markers.

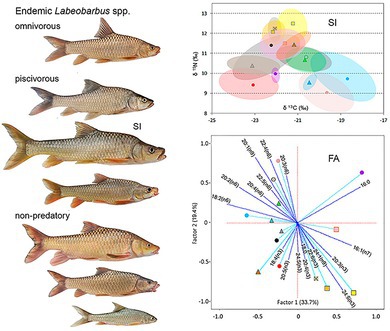

## 1 Introduction

Adaptive radiations are a manifest example for rapid speciation and such have been subject of great interest to evolutionary biologist since Simpson (Simpson 1951; Echelle and Kornfield 1984; Meyer et al. 1990; Meyer 1993; Losos 2017; Schluter 2000; McGee et al. 2020). During adaptive radiations, ecomorphs acquire phenotypic differences that are beneficial for resource partitioning and ecological specialization and can persist for generations before the ecomorphs become reproductively isolated, that is achieve species status (Meyer et al. 1990; Schluter 2000; Streelman and Danley 2003; Hernández‐Hernández 2019). Among the most prominent examples for highly diverse adaptive radiations are lacustrine‐adapted fishes (Meyer et al. 1990; Meyer 1993; Robinson and Parsons 2002; Skúlason et al. 2019). Among the textbook examples are cichlids, which radiated into hundreds of species sharing the ecological space of a single waterbody as narrowly as possible (Meyer et al. 1990; Seehausen 2006; Ronco et al. 2021). Other lineages of fish are also known to evolve divergent phenotypes and speciate in just a few thousand years, particularly in lake environments including Ethiopia's Lake Tana.

Lake Tana (12°00′ N; 37°15′ E) is situated on an ancient volcanic plateau at an elevation of 1830 m above sea level. It is rather large (3050 km^2^) but shallow (average depth 8 m, maximum 14 m). Local cyprinid assemblage of the genus *Labeobarbus* Rüppell, 1835 is evolutionarily young and represents one of the world's most famous examples of adaptive radiations (Nagelkerke et al. 1994; Mina et al. 1996; De Graaf et al. 2008). The age of diversification is only ~15 KY, when the lake last filled up and became freshwater again after repeated desiccation and salinity periods (Lamb et al. 2007; Vijverberg et al. 2009; Marshall et al. 2011). The possibly monophyletic origin of the Lake Tana labeobarbs (De Graaf et al. 2008; Levin et al. 2020) makes this assemblage an interesting and important model for studying ongoing evolutionary divergence under sympatric conditions.

Previous research identified at least fifteen endemic morphotypes of labeobarbs along with presumably ancestral generalized species with a wider distribution— *L. intermedius* Rüppell, 1835. The endemic morphotypes were described as species or reinstalled from synonyms based on their differences in functional morphology, feeding ecology, and reproductive biology (Nagelkerke and Sibbing 1997, 2000; Shkil et al. 2017). A remarkable feature of the labeobarb species flock of Lake Tana is the large number of piscivorous species, which account for almost half in the *Labeobarbus* radiation's diversity, are found in all lake habitats and differ in hunting tactics and prey capture kinematics (Nagelkerke and Sibbing 1997, 2000; De Graaf et al. 2003). However, the lack of strict reproductive isolation as shown in crossing experiments (Alekseyev et al. 1996; Dzerzhinskii et al. 2007; Shkil et al. 2017), shallow or absent genetic differences (Berrebi and Valiushok 1998; De Graaf et al. 2008; Nagelkerke et al. 2015; Beshera et al. 2023), and the partial dietary overlap among species (Nagelkerke et al. 1994; Nagelkerke and Sibbing 1997) are indicative of the early stages of their speciation, which raises questions about their morphological distinctness and ecological stability.

Earlier studies on the trophic preferences of Lake Tana labeobarbs were conducted by the Experimental Zoology Group of Wageningen University in 1990–2003, members of which have established the diet preferences and trophic specializations based on the gut content analysis (e.g., Nagelkerke 1997; Nagelkerke and Sibbing 1997). At that time, the Lake Tana ecosystem and the diversity of labeobarbs were relatively intact and not yet perturbed by human impacts on their environment. However, in the last 40 years the Lake Tana ecosystem has been subjected to pronounced human disturbance affecting its labeobarbs' populations which declined critically (Getahun et al. 2021). Anthropogenic impacts include massive water pollution, uncontrolled commercial fishing, the introduction of alien fish species, the construction of dams that block migration routes to spawning sites, and the overgrowth of river mouths by an introduced hyacinth (Nerae et al. 2024). As a result, some endemic labeobarbs of Lake Tana have been categorized as the Vulnerable (VU) or Endangered (EN) species in the IUCN Red List (https://www.iucnredlist.org/). Given these circumstances, it is unclear whether the labeobarbs' trophic niches in Lake Tana remained intact or have shifted due to adverse environmental conditions during the time since they were first studied about 40 years ago. As in the lamentable situation of the haplochromine cichlids of Lake Victoria (Witte et al. 2007) that have been threatened and many species gone extinct due to the introduction of the Nile perch and devastating anthropogenic disturbances, the Lake Tana labeobarbs are also threatened with extinction.

In late 2022, only fish with a generalized (intermediate) morphotype were collected still relatively frequently, while morphologically specialized labeobarbs were found only rarely in catches and our field work. We collected adult fish from 11 of the 15 *Labeobarbus* species that are still present in Lake Tana, in order to verify trophic divergence and to uncover current niche partitioning within the imperiled cyprinid sympatric assemblage. To address whether the initially described trophic differences remained, we evaluated two independent tropho‐ecological markers that provide a temporal‐ and spatial‐integrated representation of fish diet and habitat: muscle stable isotope (SI) ratios (Vander Zanden et al. 2006; Layman, Arrington, et al. 2007) and fatty acid (FA) compositions (Happel et al. 2016; Gladyshev et al. 2017).

## 2 Materials and Methods

### 2.1 Fish Collection

For a period of 20 days in October 2022, fish were caught in the southern part of Lake Tana and in the Gumara River, the only undammed tributary. Gillnets with a mesh size of 20 and 32 mm (150 m length) were used in the lake, while cast nets were used in the river. In addition, labeobarbs from the coastal overgrown area that has been referred to as the polymorphic and probably hybridogenic “shore (= reed) complex” (Nagelkerke et al. 1995; Mina et al. 2013) were sampled along the shoreline of Bahir‐Dar Gulf, among the reed and papyrus thickets, using gill nets (see Figure S1).

**FIGURE 1:**
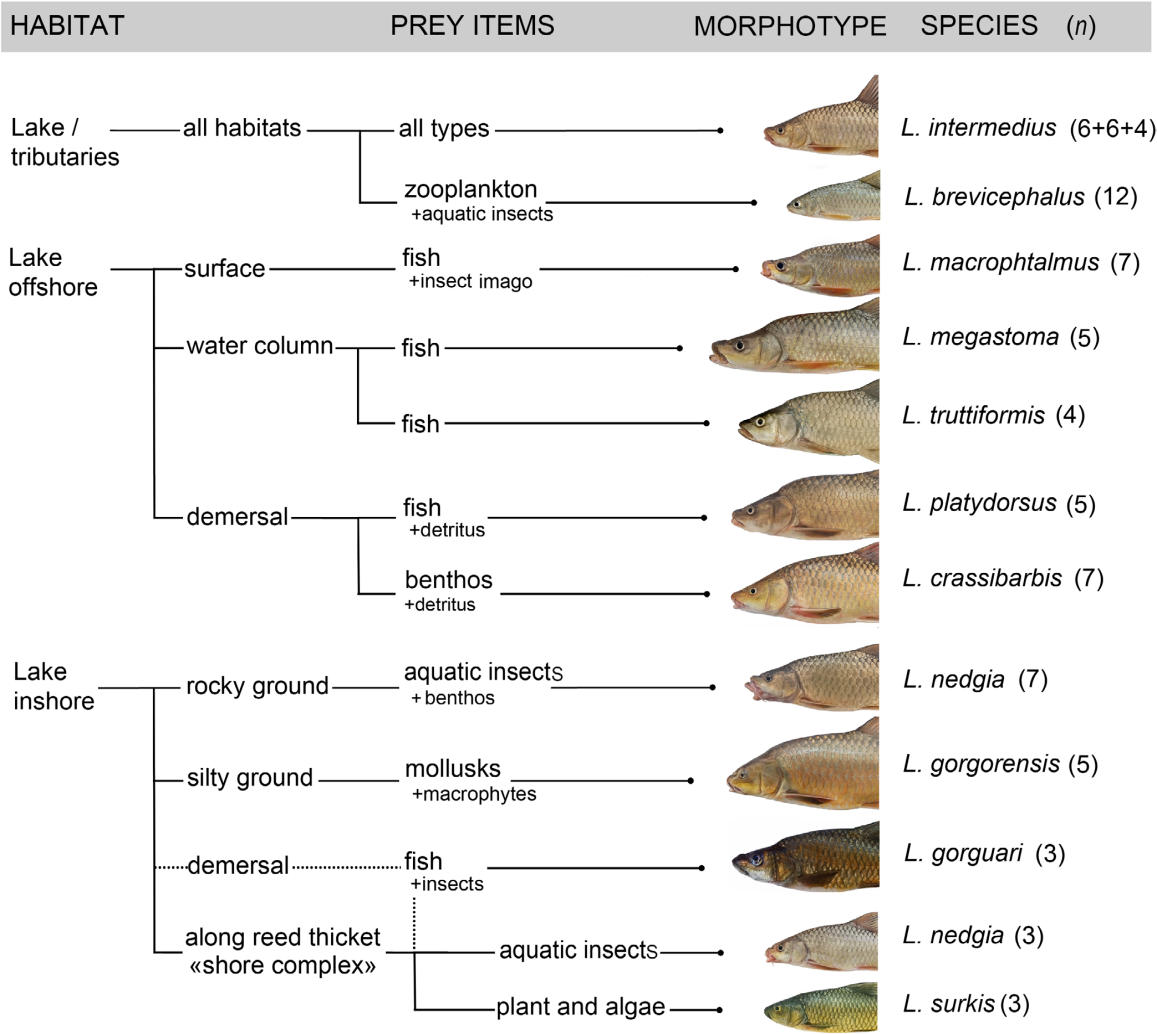
Schematic illustrating the ecological specialization and feeding preferences of *Labeobarbus* spp. caught in Lake Tana in 2022 (modified based on De Graaf et al. (2008)). Numbers in brackets indicate the number of individuals sampled and analyzed; *L. intermedius* was collected from the lake offshore as well as the tributaries and inshore (Figure S1 for additional details).

Following Nagelkerke and Sibbing (2000), who made a great effort to classify the diversity of Lake Tana labeobarbs, we used species designations for the sampled fish. In addition to the omnipresent *L. intermedius* and *L. brevicephalus*, nine species were identified in our catches: *L. gorgorensis, L. crassibarbis, L. nedgia, L. truttiformis, L. platydorsus, L. macrophtalmus*, *L. megastoma, L. gorguari*, and *L. surkis*. The first seven are off‐ and inshore dwellers. The latter two, along with the small‐sized *L. intermedius* and *L. nedgia* , represent the shore complex (Figure 1 and Figure S1 for the exterior and body size comparison). Individuals of the piscivorous *L. dainellii* and *L. acutirostris* , as well as omnivorous *L. osseensis* and *L. tsanensis*, were not found any more in 2022. Only one immature *L. longissimus* specimen was collected but not used for analysis.

Despite daily fishing and an intensive sampling effort, in total we obtained muscle samples from only 78 adult labeobarbs belonging to 11 species (Figure 1, the number of specimens used for each species is shown in brackets). To the best of our knowledge, there are no additional samples of *Labeobarbus* spp. muscle tissue of a suitable quality.

### 2.2 Fish Classification Based on Ecological Preferences

Large‐scale studies of the environmental preferences of Lake Tana labeobarbs were carried out in the 1990s using massive sampling efforts by the Dutch team from Wageningen University in The Netherlands. It was found that the *Labeobarbus* assemblage exploited all available food resources present in the ecosystem, from plants to pelagic and demersal fish. Although the trophic niches of the endemic species partially overlapped, each was shown to be characterized by a specific gut content that remained relatively stable throughout the year (Nagelkerke et al. 1994; Nagelkerke and Sibbing 1997). At that time, the species also showed relatively stable preferences in their choice of habitat across the lake (De Graaf et al. 2008). Summarizing these data with a certain degree of simplification, an approximate scheme of the environmental preferences of the originally described 14 lineages (we analyzed *L. intermedius* from the limnetic open lake, the shore complex and the tributary; as well as *L. nedgia* from the inshore region and the shore complex, separately) present in our collections has been summarized graphically (Figure 1). The distribution of fish in our catches, as well as their gut contents, is consistent with the proposed scheme.

In the present study, the expected ecological specializations of each species were analyzed based on the proposed scheme from—40 years ago—Figure 1: *L. intermedius* is a cosmopolitan omnivore, *L. macrophtalmus*, *L. megastoma* and *L. truttiformis* are pelagic piscivores, *L. platydorsus* is a demersal piscivore and detritophage; *L. crassibarbis* is a benthivore and detritophage, *L. gorgorensis* is a molluscivore, *L. brevicephalus* is a planktivore, *L. nedgia* is an insect‐feeder. The shore complex includes the small‐sized *L. intermedius* and *L. nedgia* , as well as the piscivorous *L. gorguari* and the herbivorous *L. surkis*.

### 2.3 Analysis of Stable Isotope Data

White muscle tissue from the dorsal side of the body below the dorsal fin was dried at 60°C to constant mass. The samples were weighed using a Mettler Toledo MX5 microbalance (Mettler Toledo, Columbus, OH, USA) with 2 μg accuracy and wrapped in tin capsules. The weight of the fish tissue samples varied from 250 to 500 μg. SI analysis was conducted at the Core Center of the A.N. Severtsov Institute of Ecology and Evolution RAS, Moscow. Briefly, a Thermo Delta V Plus continuous‐flow IRMS was coupled to an elemental analyzer (Flash 1112) equipped with a Thermo No‐Blank device. The isotopic composition of N and C was expressed in the δ notation relative to the international standards (atmospheric nitrogen and VPDB, respectively): δ*X* (‰) = [(Rsample/Rstandard) − 1] × 1000, where R is the molar ratio of the heavier and lighter isotopes. The samples were analyzed with a reference gas calibrated against the International Atomic Energy Agency (IAEA) reference materials USGS 40 and USGS 41 (glutamic acid). The accuracy of the measurement was ±0.2‰. The muscle lipid content was refined by extracting and evaporating lipids for fatty acid analysis (see 2.4 Section). As the lipid content was low (≤ 6% of dry weight in all groups), no arithmetic lipid correction of the isotopic ratios was performed (Post et al. 2007).

To identify the significance of isotope ratios in group differentiation, a nonparametric multidimensional PERMANOVA based on the Bray‐Curtis distance matrix was employed. *P*‐values for covariance heterogeneity were calculated based on 10,000 permutations in PAST v4.03 (Hammer et al. 2001). Species were then compared using the pairwise van der Waerden post hoc test for multiple comparisons of small samples, complemented by a Kruskal‐Wallis H test (Conover 1999). The SIBER from SIAR package (Jackson et al. 2011) in R Studio v2023.06.0 was used to examine the differences in the isotope niches. The 95% confidence ellipses were calculated for scatters in the δ13C—δ15N space (α‐level 1.0). All ellipses were adjusted to a small number of samples (SEAc—Standard Ellipse Area corrected). The percentage of overlap of the standardized ellipses was calculated. This correction is insensitive to sample size and incorporates statistical uncertainty using Bayesian approach calculations of the posterior distributions of isotope values (Swanson et al. 2015).

### 2.4 Analysis of Fatty Acid Composition

White muscle was immediately frozen at −195°C in liquid nitrogen. In laboratory, lipids were extracted from 3.0‐g minced samples in a methanol‐chloroform mixture following Folch et al. (1957). The extracts were analyzed on a Thermo DSQ II mass‐spectrometer equipped with a Thermo Focus GC gas chromatograph after acid‐catalyzed transesterification with 1% sulfuric acid in methanol (Christie and Han 2012). Butylated hydroxytoluene was added to all solvents at 100 mg/L as an antioxidant. The acids 11:0 and 19:0 were added as internal standards prior to transesterification. Separation was achieved using a SGE BPX70 column (30 m length, 0.22 mm ID, 0.25 μm film thickness) with helium as the carrier gas (constant flow mode: 0.8 mL/min). Samples were injected in split mode (1:25) at an injector temperature of 240°C. The GC oven temperature program was as follows: initial temperature of 60°C was held for 1 min; then ramped at 10°C/min to 160°C, at 2°C/min to 240 and held for 10 min. Identification was carried out by mass‐spectra, comparison with a standard mixture (Supelco 37 component FAME MIX, Sigma Aldrich) and equivalent chain length (ECL) values. Quantification ions (m/z) were 74 and 87 for saturated FA (SFA) methyl esters, 55 and 69 for monounsaturated FA (MUFA) methyl esters, 67 and 81 for dienoic FA (DUFA) methyl esters. and 67 and 79 for polyunsaturated FA (PUFA) methyl esters. GC/MS analysis was performed at the Joint Usage Center of the A.N. Severtsov Institute of Ecology and Evolution RAS, Moscow.

All acid chains with a mean relative contribution < 0.1% were filtered out of the analysis. The significance of between‐group differentiation based on the concentrations of the remaining 29 FAs (in mg g^−1^) was tested using PERMANOVA in PAST. Only FAs whose concentrations showed significant differences between species (*p* < 0.05) in the multidimensional test were included in the final dataset (18 types in total). As the dispersion of concentration values differed by more than an order of magnitude between species, the data transformation was performed for further analysis. The Centered Log‐Ratio Transformation (clr) (Filzmoser et al. 2018) was applied to the FA contributions (in %) using the *balanceBase*(x) command in R CRAN.compositions v2.0‐8 (Van den Boogaart and Tolosana‐Delgado 2013). First, we compared all species using the van der Waerden test for acid clr‐contributions. Following the proposed analysis algorithm (Greenacre 2018), we performed Canonical Variate (CV) scaling of the dataset to visualize the FA differences between species. The factorial effects of the discrimination of principal components (PC) were analyzed to evaluate the marker FAs for different species. Statsoft v10 software (Hill and Lewicki 2006) was used for the CV and PC analyses. Additionally, we analyzed the FA ratio by saturation groups, comparing the ratios of SFA, MUFA, DUFA and PUFA (expressed as the sum of the respective FA concentrations) between species using the van der Waerden test.

## 3 Results

### 3.1 Stable Isotope Composition

**FIGURE 2:**
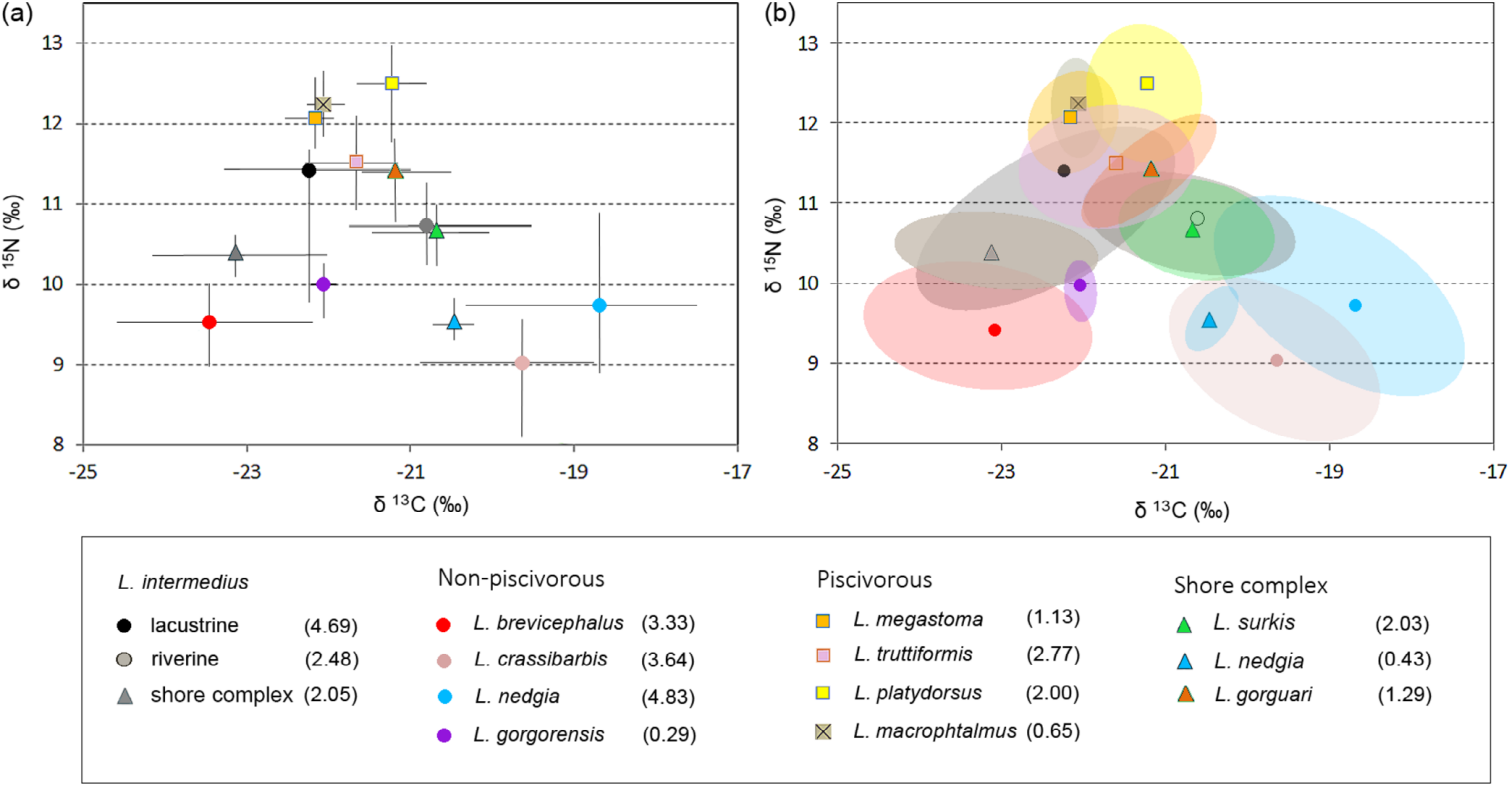
Stable isotope ratio biplot (a) showing mean values and min–max range of values, and standardized Bayesian ellipses with 95% confidence intervals (b) showing trophic niche widths and overlaps in *Labeobarbus* spp. caught in Lake Tana in 2022. Numbers in brackets in the legend indicate total convex hull areas for the groups. See Table S1 for pairwise δ^15^N and δ^13^C comparison.

Isotope ratios did not depend on fish size, Spearman *r* < 0.45 (Figure S1 for standard lengths of the analyzed individuals) suggesting no ontogenetic diet shifts, at least in our samples of mature fish. Labeobarbs were statistically differentiated by isotopic signatures, PERMANOVA *F* = 25.86 *p* < 0.001, error SS = 19,025, MS = 912 (Table 1). The range of δ^15^N/δ^13^С mean values of the species was 4.25‰/4.44‰. Piscivorous labeobarbs demonstrated a higher position in δ^15^N compared to nonpiscivorous (*p* < 0.001; Figure 2a). Among the formers, *L. platydorsus* was the most ^15^N‐enriched, while *L. gorguari* caught near the reed thickets showed a similar δ^15^N level as the omnivorous *L. intermedius* . The benthivorous *L. crassibarbis* showed the lowest δ^15^N level among the analyzed species. The planktivorous *L. brevicephalus*, as well as *L. intermedius* of the shore complex were ^13^C‐depleted, whereas the benthivorous offshore *L. nedgia* and *L. crassibarbis* were ^13^C‐enriched compared to the others (Figure 2 and Table S1).

**TABLE 1:** Permutational multivariate analysis of variance (PERMANOVA) of untransformed data.

Predictor	MS	*F*	*p* (perm)
**Stable isotopes, δ in ‰**			
^13^С	161.1	30.74	< 0.001
^13^С	170.3	32.50	< 0.001
**Fatty Acids, concentration in mkg g** ^**−1**^			
C14:0	0.1	0.04	0.849
C15:0	3.0	1.18	0.284
C16:0	11.9	4.99	0.031
C17:0	14.1	5.73	0.020
C18:0	22.2	8.88	0.004
C20:0	17.0	6.63	0.012
C16:1(n‐7)	0.7	0.27	0.605
C16:1(n‐5)	0.0	0.01	0.931
C17:1(n‐7)	2.1	0.82	0.371
C18:1(n‐7)	3.8	1.49	0.229
C16:1(n‐9)	0.1	0.05	0.830
C18:1(n‐9c)	0.1	0.05	0.825
C20:1(n‐9)	7.8	3.07	0.087
C24:1(n‐9)	26.8	11.80	0.001
C18:2(n‐6)	31.4	16.39	0.000
C20:2(n‐6)	24.3	9.72	0.002
C20:3(n‐6)	20.3	8.13	0.007
C20:4(n‐6)	12.1	4.86	0.033
C22:4(n‐6)	11.8	4.67	0.043
C22:5(n‐6)	27.2	11.90	0.001
C18:3(n‐3)	0.0	0.01	0.905
C18:4(n‐3)	11.7	4.89	0.031
C20:3(n‐3)	14.8	5.74	0.021
C20:4(n‐3)	15.9	6.11	0.018
C20:5(n‐3)	15.8	6.06	0.020
C22:5(n‐3)	1.4	0.55	0.463
C22:6(n‐3)	21.0	8.32	0.006
C24:5(n‐3)	14.6	5.80	0.018
C24:6(n‐3)	14.5	5.74	0.021

Although the results of statistical analyses based on small samples are somewhat conditional, we performed multiple pairwise comparisons between species (Table S2; *H*_14;79_ = 79.61 for δ^15^N and = 77.27 for δ^13^С; *p* < 0.001). Of 91 comparison pairs (14 groups of comparison), 57 pairs were found to be significantly different in δ^15^N and 50 pairs were found to be significantly different in δ^13^С. Particularly, the omnivorous *L. intermedius* from the lake offshore and the tributary occupied an intermediate isotopic position, differing from each other in δ^13^С (van der Waerden test *p* = 0.005) and did not differ in δ^15^N level (*p* = 1.00).

Isotopic niches, expressed as areas of Bayesian ellipses with 95% confidence intervals in δ^15^N—δ^13^С space, were widest in *L. nedgia* and *L. intermedius* from the lake offshore, and comparatively narrow in *L. gorgorensis* and inshore *L. nedgia* (Figure 2b). The most statistically isolated niches were typical for *L. crassibarbis*, *L. gorgorensis, L. brevicephalus* and *L. platydorsus* (Figure 2b and Table S3). Niche overlap (> 50%) was found between *L. truttiformis—L. megastoma*, *L. truttiformes—L. macrophtalmus*, and shore complex *L. nedgia* —inshore *L. nedgia* . Significant overlap in isotope niches (> 60.1%) was also revealed for *L. intermedius* from the lake offshore and shore complex, offshore *L. intermedius—L. truttiformis*, *L. intermedius—L. gorguari, L. truttiformis—L. gorguari* as well as between *L. crassibarbis—*inshore *L. nedgia* . We found nearly complete overlap (> 95%) in the niches of the piscivorous *L. megastoma* and *L. macrophtalmus*, as well as in the niches of the riverine *L. intermedius* and the plant‐feeding lacustrine *L. surkis* (Table S3), despite the latter pair inhabiting remote habitats.

### 3.2 Fatty Acid Composition

*Labeobarbus* muscles contained 37 fatty acids (FAs), of which only 29 exceeded 0.1% of the total FA content in all species. Of these, 18 FAs differed in their concentration between the analyzed groups (PERMANOVA *F* = 3.34 *p* = 0.003, error SS = 22.3, MS = 3.6). The concentrations of the main FAs in fish are shown in Table S4.

**FIGURE 3:**
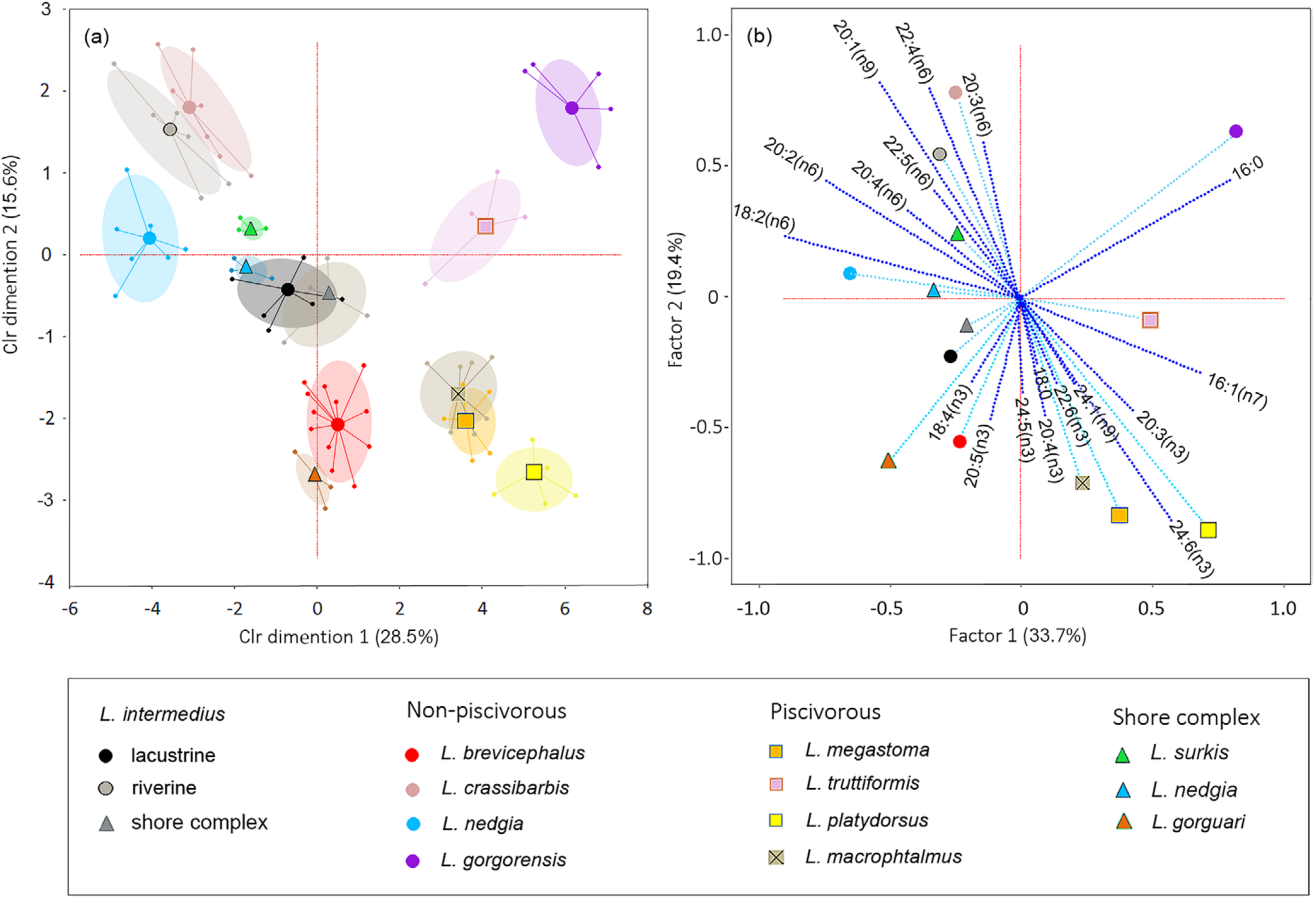
Main fatty acid log‐content biplot in CV space (a) showing confidence ellipses (95%) for *Labeobarbus* spp. caught in Lake Tana in 2022 (means are labeled, and individual points are connected to their respective means). Projection of fatty acid isometric log‐ratios onto the factor plane using PC analysis (b): The fatty acids contributing to the two‐dimensional solution of the relative position of the *Labeobarbus* spp. are labeled; the fatty acid contributions correspond to the length of the arrows from the center. The group positions in the two‐dimension factor plane are labeled; eigenvalues are % of total variance.

Most of the species were separated using the clr‐transformed individual contributions of the 18 main FAs in CV analysis (Wilks' Lambda = 0.000033, *F*_226;457_ = 3.52 *p* < 0.0001). Along the first CV axis, mainly the piscivorous species (including also the molluscivorous *L. gorgorensis*) were distinct from the nonpiscivorous labeobarbs. Along the second axis, the species that utilized food sources from the water column and the bottom were separated (Figure 3a). Offshore *L. intermedius* occupied the central position on this scale. The pairs *L. gogrorensis* vs. *L. gorguari* and *L. crassibarbis* vs. *L. platydorsus* were in opposite polar positions. Four species that comprise the shore complex were distinct and clearly identifiable with high support. However, the distances among their FA niches are, on average, twice as small as those between the offshore labeobarbs (Figure 3a). The FA niches of the pairs: *L. megastoma*—*L. macrophtalmus* and offshore *L. intermedius* —inshore *L. intermedius* overlapped almost completely.

Using PC factor scaling of the clr‐transformed FA contributions (Figure 3b), we can estimate which chains specifically mark the different paths of ecosystem transfer. These are long‐chain omega‐3 acids indicative of fish feeding, c18:4(n3) corresponding to zooplankton consumption, c18:2(n6) transferred by aquatic insects, and long‐chain omega‐6 acids indicating the benthic food chain. Moreover, factor scaling allowed the identification of FA characteristics associated with different species (Figure 3b). The piscivorous *L. macrophtalmus* and *L. megastoma* predominantly accumulated c20:4(n3) and c22:6(n3) acids. *Labeobarbus platydorsus* accumulated c24:6(n3) and *L. truttiformis—*c16:1(n7) acid. The c18:4(n3) acid was the marker for *L. gorguari* and *L. brevicephalus*, while the increased content of c20:3(n6) acid was typical for *L. crassibarbis*. Both *L. nedgia* , from the lake inshore and shore complex, accumulated c18:2(n6). The molluscivorous *L. gorgorensis* stored more c16:0 than c18:0 and unsaturated acids in muscle. *Labeobarbus surkis* and riverine *L. intermedius* , which diet includes plants, accumulated c20:1(n9) and c22:5(n6) acids. A significant increase in the contribution of the corresponding FAs was also observed when analyzing the matrix of pairwise contribution comparisons with the van der Waerden test (Table S5; the *H*‐tests for all FAs indicated *p* < 0.05).

Analyzing FAs by the ratio of SFA, MUFA, DUFA and PUFA (Table S6), we found a clear specificity of the molluscivorous *L. gorgorensis*, which differed in a significantly higher ratio of SFA (van der Waerden test *p* ≤ 0.048). The insectivorous *L. nedgia* accumulated less DUFA than others (*p* ≤ 0.001), while *L. brevicephalus* and *L. gorguari* differed in the lowest MUFA content relative to SFA (*p* ≤ 0.023). The pelagic *L. macrophtalmus* and *L. megastoma* intensively accumulated PUFA (*p* ≤ 0.024), and *L. truttiformis* accumulated SFA (*p* ≤ 0.003) like *L. gorgorensis* (*p* = 0.001).

## 4 Discussion

All *Labeobarbus* spp. are a hexaploid (2n = 150) lineage of African cyprinid fishes (Oellermann and Skelton 1990; Golubtsov and Krysanov 1993) that tend to quickly speciate both in rivers and lakes (Levin et al. 2020). Riverine adaptive radiations include from two to eight sympatric species (Levin et al. 2021, 2024), whereas the sympatric assemblage in Lake Tana was composed of up to 16 species with pronounced differences in diet specialization (Nagelkerke and Sibbing 1997, 2000; De Graaf et al. 2003). The last comprehensive study of the dietary diversity of Lake Tana species was conducted around 25 years ago based on the gut content alone, when the lake and population of labeobarbs were still intact (Nagelkerke 1997; Nagelkerke and Sibbing 2000). Since then, environmental contamination, the large‐scale exploitative fisheries on juveniles and adults, increased competition with alien species and restricted access to reproduction sites have resulted in labeobarbs populations' drastic decline (Getahun et al. 2021; Nerae et al. 2024). In light of these negative anthropogenic changes, it was unknown whether the ecological niches of the species had persisted throughout this population decline or if they had begun to respond to the human‐induced environmental disturbances. Reliable markers of trophic resource partitioning—stable isotopes (SI) and fatty acids composition (FA)—demonstrated that most species still maintain their ecological niches.

The generalized species *L. intermedius* was still present in three samples taken from the tributary, the coastal zone and the lake offshore. These fish proved to be omnivorous and occupied a central position in the SI ratio and FA composition spaces. Despite the fact that *L. intermedius* is found in different habitats, all individuals had similar diets and rather wide isotopic niches. According to the SI data, riverine *L. intermedius* exhibited similarity with *L. surkis*; FA data placed it close to *L. crassibarbis* and *L. surkis*. This suggests a shift toward an increased proportion of benthic organisms and macrophytes in its diet that of the lacustrine *L. intermedius* . Levin et al. (2024) found a similar diet of a *Labeobarbus* generalist from different Ethiopian rivers, again confirming their ancestral generalist ecology and morphology. In contrast to the trophic specialists of this species flock, *L. intermedius* could still be reliably caught in the Lake Tana basin in 2022. Therefore, one might conclude that trophic generalists may be less susceptible to extinction (or extirpation) due to their ability to switch between alternative food resources (Purvis et al. 2000).

The groups most vulnerable to severe ecosystem impacts appear to be the top predators (Layman, Quattrochi, et al. 2007). In Lake Tana these are *L. truttiformis, L. megastoma*, *L. macrophtalmus* (EN—https://www.iucnredlist.org/) and *L. platydorsus* (VU—https://www.iucnredlist.org/) (Nagelkerke and Sibbing 1997). Due to the accumulation of omega‐3 chains, which is typical of lacustrine predators, (Chavarie et al. 2016; Gladyshev et al. 2017) their FAs ratio differed significantly from that of nonpiscivorous labeobarbs. These acids are essential for maintaining rapid catabolism and high swimming ability that are indispensable for predators (Sargent et al. 2002; Schmitz and Ecker 2008). Piscivorous labeobarbs were also 2.0‰–3.5‰ higher in δ^15^N than planktivorous *L. brevicephalus* and benthivorous *L. crassibarbis*, and 1.2‰–1.5‰ higher than phytophagous *L. surkis*.

Previous analyses of nitrogen transfer through lacustrine food webs allow us to consider an approximately 2.3‰–3‰ enrichment in ^15^N as a difference between trophic levels (McCutchan Jr. et al. 2003; Vanderklift and Ponsard 2003). According to our data on the SI and FA ratios, *L. platydorsus* should have a higher proportion of fish prey in the diet than other piscivorous species. Herewith, the higher omega‐3 and omega‐6 ratio was identified for *L. megastoma*, confirming its position as top predator according to previous studies (Nagelkerke and Sibbing 1997; Sibbing and Nagelkerke 2000). Some of the piscivorous species from our catches, in particular, *L. truttiformis*, having low values of the δ^15^N in this ecological guild may have a mixed diet that included not only fish in 2022. This result contradicts data from the 1990s, when the gutSs of L. *truttiformis* were found to contain a high proportion of fish prey (Sibbing and Nagelkerke 2000). Furthermore, multidimensional scaling of FA content brought *L. truttiformis* closer to the benthivorous labeobarbs, but not *L. platydorsus*. This indicates that *L. truttiformis* is more dependent on benthic resources and makes adjustments to its ecological characteristic in Figure 1.

Both tropho‐ecological markers indicated that the ecological niches of piscivorous *L. megastoma* and *L. macrophtalmus* overlap almost completely. While these species likely feed on the same prey in the same habitat, their highly divergent phenotypes promoted the different hunting tactics, as reported by De Graaf et al. (2003). Differentiation of the ecological niches of other piscivorous species may be explained by discrepancies in preferred prey. Additional adaptive radiations of small‐sized cyprinids of the subfamily Smiliogastrinae (genus *Enteromius*) and the subfamily Labeoninae (genus *Garra*) are a unique feature of the Lake Tana ecosystem (Dejen et al. 2002). These smaller fish are preyed upon by the large *Labeobarbus* species, and this high diversity of smaller fish prey species may have led to the diversification of the piscivorous labeobarbs.

As shown in this study, the four offshore nonpiscivorous species that occupy distinct ecological niches are characterized by specific FA compositions. Fish are likely to accumulate different FAs from food items of their diet. Following the proposed scheme of the species trophic specialization (Figure 1), marker FAs can be identified for the consumers of: zooplankton—c18:4(n3) and c20:5(n3); plants—c22:5(n6); benthos and detritus—c20:3(n6) and c22:4(n6); insect larvae—c18:2(n6) acids. Given that insects are considered to be an important source of c18:2(n6) acid in freshwater ecosystems (Chavarie et al. 2016), it is worth noting that insects constitute a significant part of *L. nedgia* 's diet, similar to the diet of the analogous ‘hypertrophied‐lipped’ phenotype in some Ethiopian rivers (Levin et al. 2023). The planktivorous *L. brevicephalus* accumulates little monounsaturated FAs, while the insectivorous *L. nedgia* —diunsaturated FAs. The molluscivorous *L. gorgorensis* has the most specific FA niche. Its muscles reach of 16:0 acid (up to 27%), and all FA ratios are shifted, including the lowest proportion of PUFA.

All nonpiscivorous species occupy low δ^15^N positions, with the *L. crassibarbis* being the lowest among the Lake Tana labeobarbs. In combination with a pronounced accumulation of omega‐6 acids, a low δ^15^N position may indicate a significant role for detritus (and plant remains) in the nutrition of *L. crassibarbis*. This finding contradicts earlier analyses of *L*. crassibarbis's diet that included mainly benthos and a small amount of detritus (Nagelkerke and Sibbing 1997). The planktivorous *L. brevicephalus* and the predominantly insectivorous *L. nedgia* have extremely different carbon isotope ratios. This may indicate contrasting patterns of isotopic carbon flow along trophic chains based on water column and bottom production (Fry and Sherr 1984; Cavalli et al. 2025).

The differences between the ecological niches of the labeobarbs identified within the shore complex were smaller than the distances between the species of the lake's limnetic regions. In terms of SI and FA markers, *L. surkis* occupies a specific niche and, according to a previous study, has a high proportion of macrophytes in its diet (Nagelkerke and Sibbing 2000; De Graaf et al. 2008). However, *L. surkis* has relatively high δ^15^N values for plant feeders. Noticeably, the highly specialized periphytonophagous fish *L. beso* also displays high δ^15^N values similar to those of co‐occurring riverine piscivorous labeobarbs (Levin et al. 2021). A recent study also showed that another cypriniform fish *Oreoleuciscus potanini* (Leuciscidae) consumes macroalgae of the genus *Chara* but digests periphytic microalgae covering macroalgae, not the macroalgae itself (Dgebuadze et al. 2025). Therefore, consumption of the periphyton covering the aquatic plants may explain relatively high δ^15^N values in phytophagous *L. surkis*.

Both *L. nedgia* , that were collected from the reed thickets and open lake, were similar in the insect feeding preferences. The small‐sized inshore *L. intermedius* was very close to the offshore *L. intermedius* in its feeding spectrum as determined by both SI and FA markers. Our data showed that the inshore *L. gorguari* consumed less fish than other piscivorous species, approximately at the level of the omnivorous *L. intermedius* . At the same time, it probably consumes zooplankton, as indicated by the proximity of its FA niche and the saturated/monounsaturated FA ratio between *L. gorguari* and *L. brevicephalus*. This seems to indicate a shift in dietary preference under modern conditions. Studies from the 1990s showed that the diet of *L. gorguari* was, on average, more piscivorous than that of *L. platydorsus* and *L. macrophtalmus* (Nagelkerke 1997). At that time, juvenile labeobarbs and tilapia *Orechromis niloticus* were the main food source for *L. gorguari*.

In summary, this study demonstrates the presence of the trophic‐ecological structuring within the imperiled endemic *Labeobarbus* assemblage in Lake Tana, despite the disturbed human‐induced transformation of this ecosystem. Inferred differences between the undisturbed situation studied in the 1990s and present data might be affected by discrepancies in sample size, the size/age characteristics of the analyzed fish as well as by the ecosystem changes themselves. In particular, a drastic decline in tilapia and labeobarbs populations (Dejen et al. 2017; personal communications with commercial fishermen in 2022), whose juveniles were the significant trophic source for *L. gorguari*, may cause a shift in the diet of the latter.

While acknowledging the uncertainty due to small sample sizes, we nonetheless identified eight clearly different niches in the lake offshore (one—by generalists, four—by nonpiscivorous, and three—by piscivorous fish) that were occupied by 11 (of the 15 described) labeobarb species sampled in 2022. Among the “shore complex”, only the phytophagous *L. surkis* occupies a specific ecological niche; the other three species have a tropho‐ecological specialization similar to that of the offshore labeobarbs. Therefore, extremely fast evolved (in less than 15,000 years) the most sympatric labeobarb species still appear to maintain specific trophic‐ecological niches, even under conditions of stock decline and ongoing ecosystem restructuring. At the same time, some species display a shift in diet that may indicate a flexible response and adaptivity to an altering environment. This raises the question of whether the species of this adaptive radiation are ecologically plastic and whether their phenotypes are also plastic to be changed accordingly to their shifted diet. And if so, can we still consider them to be the same species?

## Author Contributions

**Evgeny V. Esin:** data curation (equal), formal analysis (equal), investigation (equal), methodology (equal), resources (equal), software (equal), validation (equal), visualization (equal), writing – original draft (equal). **Anastasia D. Kudryavtseva:** formal analysis (equal), investigation (equal), methodology (equal). **Boris A. Levin:** conceptualization (equal), data curation (equal), funding acquisition (equal), project administration (equal), resources (equal), writing – review and editing (equal). **Belay Abdissa:** investigation (supporting), resources (supporting), validation (supporting). **Benyam Hailu:** investigation (supporting). **Fedor N. Shkil:** data curation (supporting), investigation (equal), project administration (lead), resources (lead), validation (equal), writing – review and editing (equal). **Axel Meyer:** conceptualization (supporting), writing – review and editing (lead).

## Funding

This work was supported by Russian Science Foundation, no. 25‐14‐00124 ‘Between micro‐ and macroevolution’.

## Ethics Statement

All applicable international, national, and institutional guidelines for the care and use of animals were strictly followed. All animal procedure protocols complied with the current laws of the Federal Democratic Republic of Ethiopia and were realized under the framework of the collaboration of the Bahir‐Dar Fisheries and Other Aquatic life Research Center (BFLARC, ARARI) and Joint Ethiopian‐Russian Biological Expedition (Agreement of December 10, 2012, between the Ministry of Innovation and Technology of the Federal Democratic Republic of Ethiopia (MInT FDRE) and the Russian Academy of Sciences (RAS) on scientific and technological cooperation). The submission for publication has been approved by all relevant authors and institutions, and all persons entitled to authorship have been so named. All authors have seen and agreed to the submitted version of the manuscript.

## Conflicts of Interest

The authors declare no conflicts of interest.

## Supporting information


**Appendix S1:** ece373098‐sup‐0001‐AppendixS1.docx.

**Data S1:** ece373098‐sup‐0002‐DataS1.xls.

## Data Availability

All data including database of primary measurements are available in the Supporting Information.
