# Supplementary material for: Differentiation of Ecological Niches in Trophic Specialists From a Disturbed Lacustrine Ecosystem: Insights from the Sympatric Lake Tana Labeobarbs (Cyprinidae)
Source: Ecol Evol. 2026 Feb 13;16(2):e73098. doi: 10.1002/ece3.73098 (PMC12904841; doi:10.1002/ece3.73098)
Supplement: Supplementary file 1 — Appendix S1: ece373098‐sup‐0001‐AppendixS1.docx. [file ECE3-16-e73098-s001.docx]

SUPPLEMENTS


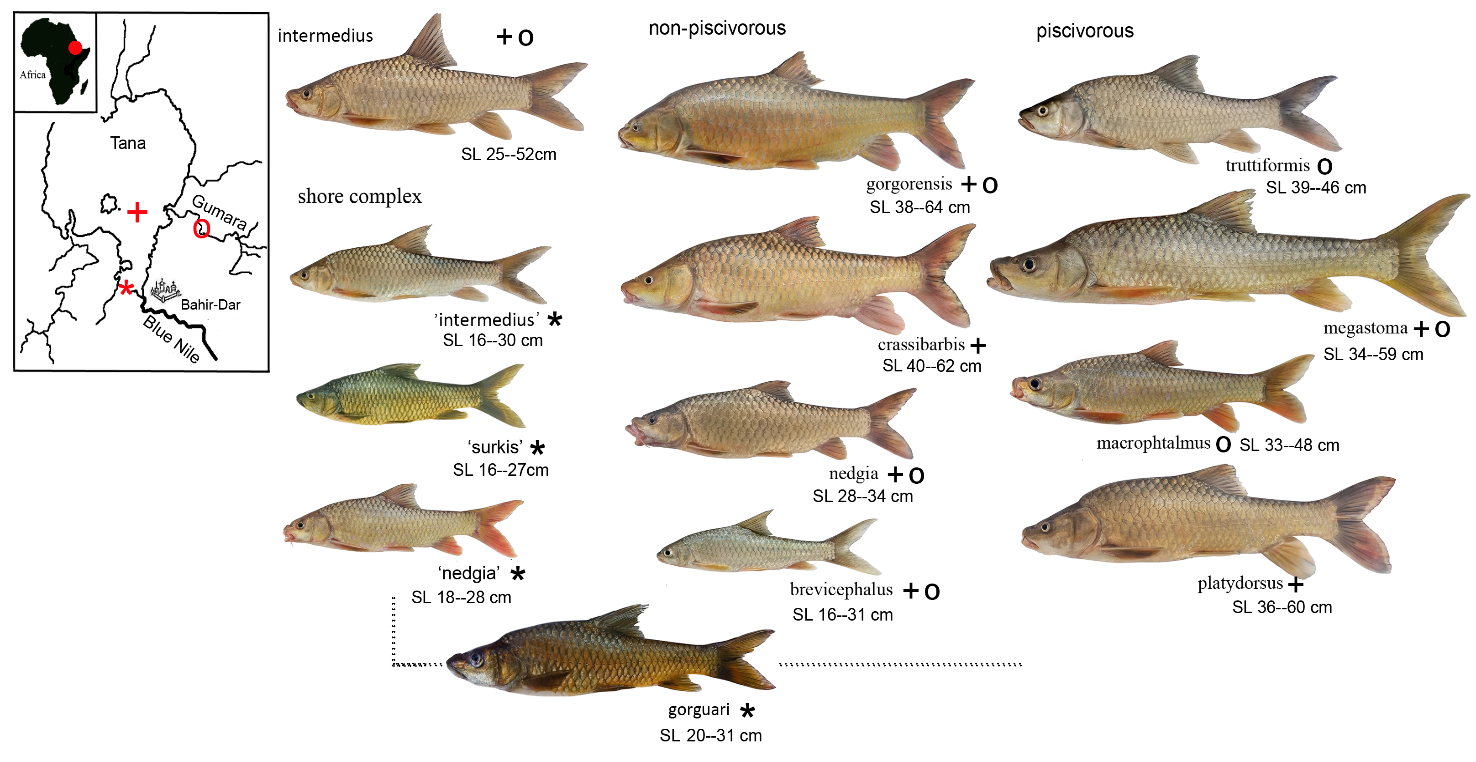


**Figure S1.** External appearance of *Labeobarbus* spp. sampled in Lake Tana in 2022 from the offshore zone (+), the lake tributary (o) and along the shoreline of Bahir-Dar Gulf (*). The standard length (SL) of the analyzed individuals is shown.

.

**Table S1.** Stable isotope ratios in the muscles of Lake Tana *Labeobarbus* spp. caught in 2022

| Groups | | N | δ^15^N | | | | | δ^13^С | | | | |
| --- | --- | --- | --- | --- | --- | --- | --- | --- | --- | --- | --- | --- |
| abbrev. | Species |  | Mean | Std. Dev | Min | - | Max | Mean | Std. Dev | Min | - | Max |
| lacustrine | *L. intermedius* | 6 | 11.2 | 0.61 | 9.8 | - | 11.5 | -22.1 | 0.82 | -23.3 | - | -21.0 |
| riverine | *L. intermedius* | 6 | 10.8 | 0.35 | 10.2 | - | 11.3 | -20.7 | 0.80 | -21.7 | - | -19.5 |
| NE | *L. nedgia* | 7 | 9.7 | 0.74 | 8.9 | - | 11.0 | -18.7 | 0.86 | -20.3 | - | -17.5 |
| GO | *L. gorgorensis* | 5 | 10.0 | 0.24 | 9.6 | - | 10.3 | -22.1 | 0.11 | -22.2 | - | -21.9 |
| shore IN | *L. intermedius* | 4 | 10.4 | 0.19 | 10.1 | - | 10.5 | -23.1 | 0.87 | -24.1 | - | -22.0 |
| shore NE | *L. nedgia* | 3 | 9.5 | 0.21 | 9.3 | - | 9.8 | -20.5 | 0.17 | -20.6 | - | -20.2 |
| shore ZU | *L. surkis* | 3 | 10.7 | 0.29 | 10.3 | - | 11.0 | -20.7 | 0.65 | -21.5 | - | -20.0 |
| BR | *L. brevicephalus* | 12 | 9.5 | 0.31 | 9.0 | - | 10.0 | -23.1 | 0.59 | -24.6 | - | -22.2 |
| CR | *L. crassibarbis* | 7 | 9.0 | 0.53 | 8.1 | - | 9.6 | -19.6 | 0.66 | -20.9 | - | -18.8 |
| shore GR | *L. gorguari* | 3 | 11.4 | 0.49 | 10.7 | - | 11.8 | -21.2 | 0.50 | -21.6 | - | -20.5 |
| MA | *L. macrophtalmus* | 7 | 12.2 | 0.25 | 11.8 | - | 12.6 | -22.1 | 0.13 | -22.2 | - | -21.9 |
| ME | *L. megastoma* | 5 | 12.1 | 0.44 | 11.6 | - | 12.6 | -22.1 | 0.24 | -22.5 | - | -21.9 |
| TY | *L. truttiformis* | 4 | 11.5 | 0.52 | 10.9 | - | 12.0 | -21.7 | 0.57 | -22.3 | - | -21.0 |
| PL | *L. platydorsus* | 5 | 12.5 | 0.48 | 11.8 | - | 13.0 | -21.2 | 0.35 | -21.7 | - | -20.8 |

**Table S2.** Significance level of difference (*p*) derived from pairwise van der Waerden test for δ ^15^N (above) and δ ^13^C (below) isotopes value comparison among Lake Tana *Labeobarbus* spp. caught in 2022

| Groups  of comparison  abbrev. Species | | Omnivorous | | Non-piscivorous | | | | |  | | Piscivorous | | | | | |
| --- | --- | --- | --- | --- | --- | --- | --- | --- | --- | --- | --- | --- | --- | --- | --- | --- |
|  |  | lac | riv | NE | GO | sIN | sNE | sZU | BR | CR | sGR | MA | ME | TR | PL |  |
| lacustrine | *L. intermedius* |  | 1.000 | **0.000** | **0.002** | 0.729 | **0.000** | 1.000 | **0.000** | **0.000** | 1.000 | **0.001** | **0.006** | 1.000 | **0.000** |  |
| riverine | *L. intermedius* | **0.005** |  | **0.002** | 0.311 | 1.000 | **0.003** | 1.000 | **0.000** | **0.000** | 1.000 | **0.000** | **0.000** | 0.896 | **0.000** |  |
| NE | *L. nedgia* | **0.000** | **0.000** |  | 1.000 | 1.000 | 1.000 | **0.014** | 1.000 | **0.032** | **0.000** | **0.000** | **0.000** | **0.000** | **0.000** |  |
| GO | *L. gorgorensis* | 1.000 | **0.011** | **0.000** |  | 1.000 | 1.000 | 0.058 | 1.000 | **0.009** | **0.001** | **0.000** | **0.000** | **0.000** | **0.000** |  |
| shore IN | *L. intermedius* | 0.654 | **0.000** | **0.000** | 0.713 |  | 0.759 | 1.000 | **0.050** | **0.000** | 0.194 | **0.000** | **0.000** | **0.050** | **0.000** |  |
| shore NE | *L. nedgia* | **0.005** | 1.000 | **0.001** | **0.010** | **0.000** |  | **0.010** | 1.000 | 1.000 | **0.000** | **0.000** | **0.000** | **0.000** | **0.000** |  |
| shore ZU | *L. surkis* | **0.014** | 1.000 | **0.000** | **0.026** | **0.000** | 1.000 |  | **0.000** | **0.000** | 0.057 | **0.000** | **0.001** | 0.052 | **0.000** |  |
| BR | *L. brevicephalus* | **0.038** | **0.000** | **0.000** | 0.057 | 1.000 | **0.000** | **0.000** |  | 1.000 | **0.000** | **0.000** | **0.000** | **0.000** | **0.000** |  |
| CR | *L. crassibarbis* | **0.000** | 0.117 | 0.258 | **0.000** | **0.000** | 1.000 | 0.381 | **0.000** |  | **0.000** | **0.000** | **0.000** | **0.000** | **0.000** |  |
| shore GR | *L. gorguari* | 1.000 | 1.000 | **0.000** | 1.000 | **0.002** | 1.000 | 1.000 | **0.000** | **0.006** |  | 0.087 | 1.000 | 1.000 | **0.025** |  |
| MA | *L. macrophtalmus* | 1.000 | **0.003** | **0.000** | 1.000 | 0.479 | **0.004** | **0.011** | **0.018** | **0.000** | 1.000 |  | 1.000 | 0.385 | 1.000 |  |
| ME | *L. megastoma* | 1.000 | **0.011** | **0.000** | 1.000 | 1.000 | **0.010** | **0.026** | **0.022** | **0.000** | 1.000 | 1.000 |  | 1.000 | 1.000 |  |
| TY | *L. truttiformis* | 1.000 | 0.063 | **0.000** | 1.000 | **0.048** | 0.206 | 1.000 | **0.001** | **0.000** | 1.000 | 1.000 | 1.000 |  | **0.027** |  |
| PL | *L. platydorsus* | 0.712 | 1.000 | **0.000** | 1.000 | **0.000** | 1.000 | 1.000 | **0.000** | **0.001** | 1.000 | **0.046** | **0.044** | 1.000 |  |  |

Note. Significant values in bold

**Table S3.** Standardized isotope niche overlap (% of posterior probability) derived from SIBER calculations with SEA correction for Lake Tana *Labeobarbus* spp. caught in 2022

| Groups of comparison  abbrev. Group | | Omnivorous | | Non-piscivorous | | | | |  | | Piscivorous | | | | | |
| --- | --- | --- | --- | --- | --- | --- | --- | --- | --- | --- | --- | --- | --- | --- | --- | --- |
|  |  | lac | riv | NE | GO | sIN | sNE | sZU | BR | CR | sGR | MA | ME | TR | PL |  |
| lacustrine | *L. intermedius* |  | 44.3 | 26.2 | 32.2 | **78.9** | 19.0 | 18.5 | 33.7 | 14.9 | **60.6** | 35.5 | 40.6 | **78.5** | 24.8 |  |
| riverine | *L. intermedius* |  |  | 35.8 | 17.4 | 42.2 | 23.5 | **96.3** | 12.3 | 24.5 | 48.3 | 15.9 | 19.3 | 39.5 | 17.0 |  |
| NE | *L. nedgia* |  |  |  | 12.4 | 16.6 | **51.8** | 40.6 | 7.7 | 49.6 | 18.4 | 6.5 | 9.4 | 20.9 | 5.5 |  |
| GO | *L. gorgorensis* |  |  |  |  | 35.5 | 20.7 | 13.3 | 16.1 | 11.5 | 14.5 | 6.4 | 8.3 | 16.8 | 3.2 |  |
| shore IN | *L. intermedius* |  |  |  |  |  | 23.2 | 18.9 | 26.5 | 11.9 | 16.0 | 9.6 | 11.4 | 23.9 | 9.7 |  |
| shore NE | *L. nedgia* |  |  |  |  |  |  | 13.5 | 8.9 | **61.8** | 4.5 | 2.0 | 2.8 | 5.5 | 3.7 |  |
| shore ZU | *L. surkis* |  |  |  |  |  |  |  | 10.5 | 21.6 | 38.6 | 16.8 | 22.8 | 32.4 | 28.8 |  |
| BR | *L. brevicephalus* |  |  |  |  |  |  |  |  | 6.3 | 5.5 | 2.4 | 2.4 | 5.9 | 1.9 |  |
| CR | *L. crassibarbis* |  |  |  |  |  |  |  |  |  | 4.7 | 1.5 | 1.7 | 6.2 | 1.9 |  |
| shore GR | *L. gorguari* |  |  |  |  |  |  |  |  |  |  | 23.1 | 30.4 | **74.0** | 42.8 |  |
| MA | *L. macrophtalmus* |  |  |  |  |  |  |  |  |  |  |  | **95.4** | **51.6** | 23.7 |  |
| ME | *L. megastoma* |  |  |  |  |  |  |  |  |  |  |  |  | **57.9** | 40.3 |  |
| TR | *L. truttiformis* |  |  |  |  |  |  |  |  |  |  |  |  |  | 39.2 |  |
| PL | *L. platydorsus* |  |  |  |  |  |  |  |  |  |  |  |  |  |  |  |

Note. Significant overlaps are highlighted

**Table S4.** Concentrations of main fatty acids (Mean, mg g^-1^ and Std. Dev.) in the muscles of Lake Tana *Labeobarbus* spp. caught in 2022

| Fatty  acid | Omnivorous | | Non-piscivorous | | | | | | Piscivorous | | | | |
| --- | --- | --- | --- | --- | --- | --- | --- | --- | --- | --- | --- | --- | --- |
|  | lac  (6 spec.) | riv  (6 spec.) | NE  (7 spec.) | GO  (5 spec.) | sIN  (4 spec.) | sZU  (3 spec.) | BR  (12 spec.) | CR  (7 spec.) | MA  (7 spec.) | ME  (5 spec.) | TR  (4 spec.) | PL  (5 spec.) | sGR  (3 spec.) |
| 16:00 | 204 | 103 | 291 | 1078 | 501 | 98 | 512 | 260 | 273 | 231 | 803 | 501 | 252 |
|  | 41.8 | 26.4 | 94.1 | 512.1 | 327.5 | 21.1 | 232.7 | 119.4 | 140.8 | 76.0 | 214.2 | 385.5 | 51.7 |
| 18:00 | 141 | 73 | 195 | 320 | 214 | 70 | 238 | 180 | 206 | 129 | 407 | 235 | 178 |
|  | 15.1 | 38.7 | 57.1 | 142.8 | 100.7 | 33.3 | 62.5 | 61.6 | 50.9 | 36.0 | 88.0 | 92.5 | 55.2 |
| 16:1(n7) | 49 | 13 | 30 | 259 | 102 | 10 | 218 | 20 | 105 | 18 | 428 | 186 | 15 |
|  | 33.1 | 3.8 | 15.1 | 114.4 | 94.0 | 3.5 | 192.2 | 12.0 | 88.2 | 6.2 | 160.2 | 224.6 | 3.3 |
| 20:1(n9) | 9.3 | 15.5 | 23.1 | 108.3 | 19.8 | 16.1 | 10.7 | 25.5 | 20.2 | 7.7 | 38.8 | 22.6 | 5.8 |
|  | 3.57 | 12.23 | 19.28 | 68.88 | 18.34 | 9.53 | 5.38 | 25.00 | 9.84 | 3.27 | 8.33 | 18.52 | 2.51 |
| 24:1(n9) | 2.7 | 0.9 | 3.1 | 7.5 | 3.4 | 1.1 | 3.4 | 3.9 | 6.6 | 5.5 | 12.9 | 5.0 | 5.6 |
|  | 1.99 | 0.90 | 0.81 | 0.91 | 2.22 | 0.83 | 1.79 | 2.81 | 0.95 | 1.14 | 2.64 | 0.79 | 1.32 |
| 18:2(n6) | 26.2 | 17.5 | 213.6 | 45.6 | 93.1 | 15.8 | 56.9 | 77.8 | 36.2 | 52.4 | 73.4 | 54.9 | 41.3 |
|  | 16.58 | 9.66 | 40.12 | 23.06 | 54.07 | 9.26 | 23.73 | 62.16 | 11.80 | 41.84 | 22.22 | 30.60 | 28.30 |
| 20:2(n6) | 1.3 | 5.3 | 9.4 | 14.6 | 3.0 | 5.1 | 0.8 | 8.4 | 1.5 | 2.3 | 5.6 | 2.7 | 1.6 |
|  | 0.73 | 3.73 | 4.84 | 4.31 | 1.71 | 2.99 | 0.66 | 4.48 | 0.93 | 1.79 | 2.23 | 2.32 | 0.31 |
| 20:3(n6) | 5.2 | 2.9 | 15.5 | 6.1 | 6.9 | 2.8 | 3.7 | 26.8 | 3.2 | 2.3 | 6.6 | 2.9 | 2.1 |
|  | 3.70 | 2.22 | 2.37 | 2.21 | 4.48 | 2.16 | 1.66 | 13.14 | 0.97 | 1.10 | 1.96 | 1.75 | 1.10 |
| 20:4(n6) | 137 | 132 | 212 | 277 | 164 | 140 | 183 | 201 | 129 | 129 | 245 | 59 | 158 |
|  | 51.5 | 36.2 | 64.6 | 51.6 | 69.3 | 29.6 | 45.9 | 31.4 | 22.0 | 42.5 | 32.0 | 25.0 | 39.4 |
| 22:4(n6) | 13.8 | 7.4 | 17.8 | 65.2 | 11.9 | 7.9 | 8.7 | 32.6 | 14.6 | 9.0 | 27.3 | 10.4 | 10.9 |
|  | 7.27 | 6.14 | 10.04 | 21.84 | 5.92 | 6.19 | 3.27 | 14.30 | 3.08 | 2.38 | 7.09 | 5.85 | 3.62 |
| 22:5(n6) | 29.9 | 20.3 | 18.2 | 99.2 | 34.1 | 22.0 | 19.3 | 24.0 | 49.1 | 32.6 | 113.8 | 42.6 | 21.8 |
|  | 8.41 | 11.45 | 16.48 | 24.59 | 17.13 | 12.33 | 9.05 | 8.27 | 7.36 | 7.51 | 44.68 | 14.55 | 3.76 |
| 18:4(n3) | 0.9 | 0.4 | 1.3 | 6.9 | 4.3 | 0.6 | 13.0 | 0.3 | 2.8 | 0.7 | 18.1 | 3.3 | 0.0 |
|  | 0.97 | 0.39 | 1.65 | 4.23 | 4.45 | 0.70 | 9.78 | 0.58 | 1.81 | 1.13 | 7.81 | 1.76 | 0.00 |
| 20:3(n3) | 3.6 | 2.3 | 6.2 | 7.9 | 5.8 | 2.5 | 5.7 | 3.7 | 5.3 | 4.7 | 10.5 | 3.5 | 3.0 |
|  | 1.53 | 1.41 | 1.83 | 2.14 | 5.25 | 1.21 | 2.17 | 1.44 | 2.04 | 1.87 | 3.59 | 1.73 | 0.28 |
| 20:4(n3) | 1.7 | 1.4 | 1.3 | 5.8 | 6.7 | 1.3 | 10.3 | 2.1 | 7.6 | 2.6 | 23.3 | 3.5 | 0.6 |
|  | 0.94 | 1.24 | 1.24 | 3.33 | 6.96 | 1.13 | 6.48 | 0.73 | 3.84 | 2.83 | 10.31 | 2.70 | 0.52 |
| 20:5(n3) | 79 | 37 | 89 | 156 | 150 | 39 | 250 | 56 | 132 | 67 | 281 | 140 | 67 |
|  | 12.7 | 21.1 | 42.7 | 45.7 | 149.2 | 23.6 | 96.0 | 14.7 | 28.8 | 24.8 | 55.3 | 71.6 | 14.0 |
| 22:6(n3) | 375 | 206 | 306 | 660 | 526 | 222 | 530 | 298 | 601 | 451 | 1280 | 610 | 498 |
|  | 36.1 | 101.0 | 55.9 | 175.6 | 302.7 | 89.6 | 170.3 | 88.0 | 169.4 | 108.8 | 238.3 | 316.7 | 99.6 |
| 24:5(n3) | 1.0 | 0.3 | 0.9 | 4.4 | 5.2 | 0.5 | 5.1 | 0.8 | 4.1 | 1.8 | 17.1 | 4.8 | 2.1 |
|  | 1.12 | 0.48 | 1.14 | 1.94 | 5.92 | 0.28 | 2.83 | 0.80 | 1.52 | 0.85 | 8.15 | 5.07 | 1.34 |
| 24:6(n3) | 12.7 | 2.7 | 9.7 | 7.8 | 15.4 | 3.1 | 9.7 | 8.8 | 5.7 | 5.1 | 14.1 | 5.8 | 5.2 |
|  | 6.03 | 1.52 | 3.53 | 2.36 | 12.50 | 1.32 | 4.98 | 2.68 | 1.36 | 2.87 | 9.85 | 3.80 | 0.73 |

(abbreviations of group names similar to Table S1)

**Table S5.** Groups that significantly differed based on van der Waerden tests for clr-transformed fatty acid contributions of Lake Tana *Labeobarbus* spp. caught in 2022

| Fatty | Omnivorous | | Non-piscivorous | | | | | | | Piscivorous | | | | |
| --- | --- | --- | --- | --- | --- | --- | --- | --- | --- | --- | --- | --- | --- | --- |
| Acid | lac | riv | NE | GO | sIN | sNE | sZU | BR | CR | MA | ME | TR | PL | sGR |
| 16:0 | 0.41 | 0.21 | 0.58 | 2.16 | 1.00 | 0.05 | 0.50 | 1.02 | 0.52 | 0.55 | 0.46 | 1.61 | 1.00 | 0.50 |
| 18:0 | 0.71 | 0.69 | 0.68 | 0.30 | 0.51 | 0.50 | 0.66 | 0.53 | 0.64 | 0.92 | 0.59 | 0.56 | 0.61 | 0.70 |
| 16:1(n7) | 0.28 | 0.13 | 0.10 | 0.24 | 0.16 | 0.22 | 0.40 | 0.39 | 0.08 | 0.50 | 0.08 | 0.60 | 0.28 | 0.06 |
| 20:1(n9) | 0.05 | 0.14 | 0.07 | 0.10 | 0.04 | 0.09 | 0.11 | 0.02 | 0.09 | 0.09 | 0.04 | 0.05 | 0.04 | 0.02 |
| 24:1(n9) | 0.01 | 0.01 | 0.01 | 0.01 | 0.01 | 0.01 | 0.01 | 0.01 | 0.01 | 0.03 | 0.03 | 0.02 | 0.01 | 0.02 |
| 18:2(n6) | 0.12 | 0.17 | 0.77 | 0.04 | 0.28 | 0.59 | 0.26 | 0.12 | 0.30 | 0.16 | 0.24 | 0.10 | 0.16 | 0.16 |
| 20:2(n6) | 0.01 | 0.05 | 0.03 | 0.01 | 0.01 | 0.02 | 0.02 | 0.00 | 0.03 | 0.01 | 0.01 | 0.01 | 0.01 | 0.01 |
| 20:3(n6) | 0.03 | 0.03 | 0.06 | 0.01 | 0.02 | 0.02 | 0.10 | 0.01 | 0.12 | 0.01 | 0.01 | 0.01 | 0.01 | 0.01 |
| 20:4(n6) | 0.83 | 0.57 | 0.78 | 0.29 | 0.46 | 0.51 | 0.60 | 0.40 | 0.94 | 0.57 | 0.58 | 0.33 | 0.37 | 0.63 |
| 22:4(n6) | 0.07 | 0.07 | 0.07 | 0.06 | 0.03 | 0.06 | 0.11 | 0.02 | 0.16 | 0.06 | 0.04 | 0.04 | 0.03 | 0.04 |
| 22:5(n6) | 0.15 | 0.17 | 0.07 | 0.10 | 0.09 | 0.09 | 0.15 | 0.05 | 0.15 | 0.11 | 0.14 | 0.13 | 0.11 | 0.09 |
| 18:4(n3) | 0.00 | 0.00 | 0.01 | 0.01 | 0.01 | 0.01 | 0.01 | 0.03 | 0.00 | 0.01 | 0.00 | 0.02 | 0.01 | 0.00 |
| 20:3(n3) | 0.02 | 0.02 | 0.02 | 0.01 | 0.01 | 0.02 | 0.02 | 0.01 | 0.02 | 0.02 | 0.02 | 0.01 | 0.03 | 0.01 |
| 20:4(n3) | 0.01 | 0.01 | 0.01 | 0.01 | 0.01 | 0.01 | 0.01 | 0.02 | 0.01 | 0.03 | 0.01 | 0.03 | 0.01 | 0.00 |
| 20:5(n3) | 0.40 | 0.37 | 0.34 | 0.16 | 0.30 | 0.35 | 0.39 | 0.52 | 0.27 | 0.59 | 0.30 | 0.38 | 0.33 | 0.25 |
| 22:6(n3) | 1.92 | 1.99 | 1.13 | 0.67 | 1.17 | 1.17 | 1.55 | 1.17 | 1.32 | 2.65 | 2.06 | 1.72 | 2.14 | 1.98 |
| 24:5(n3) | 0.01 | 0.01 | 0.00 | 0.01 | 0.01 | 0.01 | 0.01 | 0.03 | 0.01 | 0.03 | 0.01 | 0.02 | 0.01 | 0.01 |
| 24:6(n3) | 0.02 | 0.03 | 0.04 | 0.01 | 0.03 | 0.03 | 0.04 | 0.02 | 0.03 | 0.04 | 0.05 | 0.03 | 0.06 | 0.03 |

Note. Clr-transformed values are shown. Colour filling indicates significant difference from mean value according to van der Waerden test:

|  | - much less (p < 0.01) |  | - less (0.01< p< 0.05) |  | - more (0.01< p < 0.05) |  | - much more (p < 0.01). |
| --- | --- | --- | --- | --- | --- | --- | --- |

(abbreviations of group names similar to Table S1)

**Table S6.** Ratio of fatty acids in the muscles of Lake Tana *Labeobarbus* spp. caught in 2022, and results of the ratio comparison using the van der Waerden test

| Groups of comparison | | Fatty acid groups | | | |
| --- | --- | --- | --- | --- | --- |
| abbrev. | group | SFA/PUFA  (H_14;79_ = 3.9 p < 0.001) | SFA/MUFA  (H_14;79_ = 3.4 p = 0.001) | SFA/DUFA  (H_14;79_ = 15.8 p < 0.001) | MUFA/PUFA  (H_14;79_ = 3.1 p=0.005 |
| lac | *L. intermedius* | 0.55 | 1.49 | 13.86 | 0.39 |
| riv | *L. intermedius* | 0.66 | 1.44 | 12.64 | 0.50 |
| NE | *L. nedgia* | 0.65 | 1.24 | 3.97 | 0.61 |
| GO | *L. gorgorensis* | 0.88 | 0.93 | 16.84 | 1.02 |
| sIN | *L. intermedius* | 0.73 | 1.46 | 9.53 | 0.56 |
| sNE | *L. nedgia* | 0.66 | 1.43 | 6.65 | 0.66 |
| sZU | *L. surkis* | 0.65 | 1.37 | 6.93 | 0.68 |
| BR | *L. brevicephalus* | 0.63 | 1.64 | 14.48 | 0.47 |
| CR | *L. crassibarbis* | 0.69 | 1.29 | 6.77 | 0.75 |
| sGR | *L. gorguari* | 0.59 | 1.91 | 13.30 | 0.58 |
| MA | *L. macrophtalmus* | 0.53 | 1.08 | 14.30 | 0.28 |
| ME | *L. megastoma* | 0.50 | 1.46 | 11.45 | 0.32 |
| TR | *L. truttiformis* | 0.65 | 1.01 | 16.18 | 0.66 |
| PL | *L. platydorsus* | 0.59 | 1.14 | 14.83 | 0.59 |

Note. Colour filling indicates significant difference from mean value according to van der Waerden test:

|  | - less (p< 0.05) |  | - more (p < 0.05) |
| --- | --- | --- | --- |
